# Eosinophilic Pneumonia Triggered by *Toxocara canis* in a Patient with Primary Ciliary Dyskinesia: A Clinical Case Report

**DOI:** 10.3390/medicina60111874

**Published:** 2024-11-15

**Authors:** Kacper Packi, Wanda Fugiel, Violetta Gołąbek, Alicja Rudek, Agnieszka Śliwińska

**Affiliations:** 1 Department of Nucleic Acid Biochemistry, Medical University of Lodz, 92-213 Lodz, Poland; agnieszka.sliwinska@umed.lodz.pl; 2 AllerGen Center of Personalized Medicine, 97-300 Piotrkow Trybunalski, Poland; wanda.jank@gmail.com (W.F.); alicjarudek@o2.pl (A.R.); 3 Wladyslaw Bieganski Collegium Medicum, Jan Dlugosz University in Czestochowa, 42-200 Czestochowa, Poland

**Keywords:** eosinophilic pneumonia, *Toxocara canis*, Kartagener syndrome, dyskinesia

## Abstract

Primary ciliary dyskinesia (PCD) is a rare genetic disorder that affects the structure and function of cilia, primarily impacting the respiratory system. Kartagener syndrome, a subset of PCD, is characterized by situs inversus, bronchiectasis, and chronic sinusitis. Patients with PCD are prone to recurrent respiratory infections due to impaired ciliary function, which hinders effective mucus clearance and promotes pathogen colonization. This case report describes a 24-year-old woman with congenital Kartagener syndrome who developed eosinophilic pneumonia caused by *Toxocara canis*, a rare parasitic infection that less commonly affects the lungs. Despite initial treatment for a presumed bacterial infection, the patient’s symptoms persisted. Further diagnostics revealed elevated eosinophil counts, total IgE, and the presence of *Toxocara canis* antibodies. The patient was treated with albendazole, resulting in significant symptom improvement and a reduction in inflammatory markers. This case underscores the diagnostic challenges in treating PCD patients, where atypical infections must be considered, particularly when standard treatments prove ineffective. The complexity of the patient’s condition required interdisciplinary management, integrating parasitological, immunological, and respiratory expertise to ensure appropriate treatment. The case highlights the need for further research into the interactions between congenital respiratory disorders such as Kartagener syndrome and parasitic infections. It also emphasizes the importance of a comprehensive diagnostic approach in managing rare genetic diseases complicated by opportunistic infections. Early detection of parasitic infections in PCD patients is crucial to preventing severe complications, and this case reinforces the necessity of considering parasitic causes in atypical pneumonia cases.

## 1. Introduction

**Figure 1:**
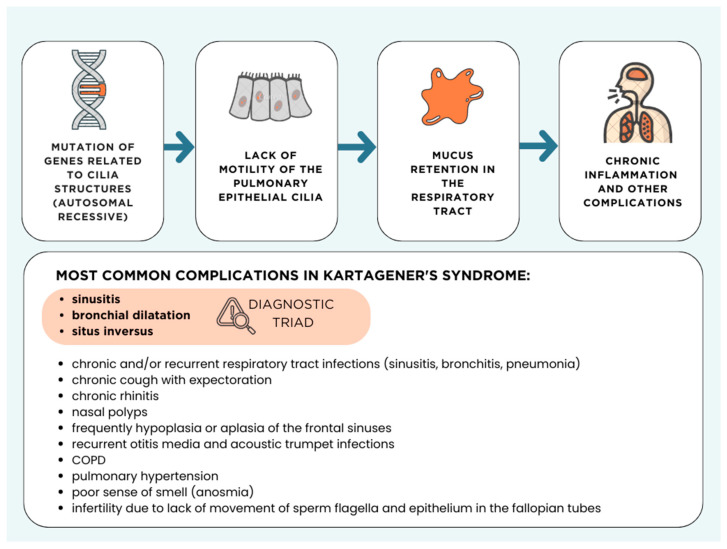
Etiology and complications of Kartagener’s syndrome.

Primary ciliary dyskinesia (PCD) is a rare, genetically conditioned disease affecting 1 in 10,000 to 20,000 live births, depending on the studied population [1]. In Europe and North America, the incidence of PCD varies depending on genetic background and demographic factors. This disease is characterized by the abnormal structure and/or function of cilia, leading to a range of disorders in the body, particularly in the respiratory system (Figure 1). Kartagener syndrome, which accounts for about 50% of all PCD cases, is distinguished by a characteristic triad of symptoms: situs inversus, bronchiectasis, and chronic sinusitis [2].

**Figure 2:**
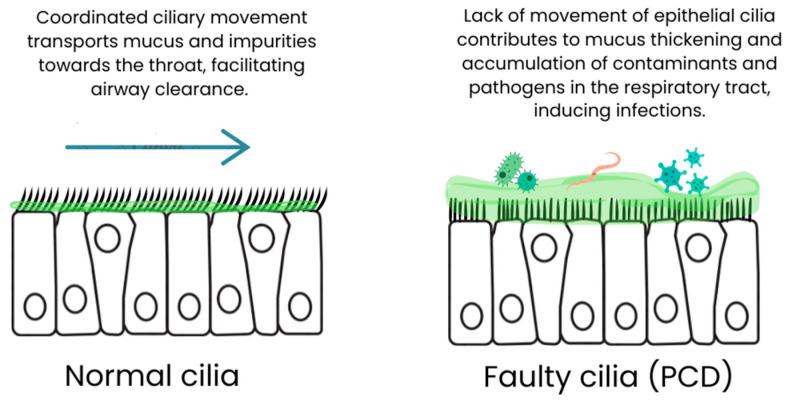
The role of epithelial ciliary movement in airway clearance.

Cilia, microscopic hair-like structures, line the cells of the respiratory tract and play a key role in the mechanical defense of the respiratory system. Under physiological conditions, their coordinated movement enables the efficient clearance of the airways by transporting mucus along with impurities toward the throat. Disturbances in ciliary movement or a complete lack thereof, observed in PCD, lead to mucus accumulation, significantly increasing the risk of recurrent infections and inflammation [3] (Figure 2). The impaired function of cilia in PCD has far-reaching consequences for the respiratory system. Impaired airway clearance promotes colonization by pathogens, primarily bacteria but also viruses and fungi. The accumulated mucus becomes an excellent medium for microorganisms, leading to chronic and recurrent infections [4]. Furthermore, the accumulation of impurities and cellular debris can trigger and sustain inflammation, contributing to progressive lung tissue damage. Moreover, ciliary movement disorders can affect the local immune response. Studies suggest that cilia play a role in regulating immune responses, and their dysfunction may lead to abnormalities in the body’s defense reactions [5]. This, in turn, can further increase susceptibility to infections and complicate the course of the disease.

In the context of PCD, infections caused by pathogens that typically rarely affect the lungs are particularly interesting. In individuals with PCD, micro- and macroorganisms such as parasites, which are normally efficiently eliminated from the respiratory tract, can more easily penetrate, accumulate, and multiply in lung tissue. The increased susceptibility to atypical lung infections in PCD patients poses an additional diagnostic and therapeutic challenge for clinicians. Overlapping symptoms and the need for differential diagnosis require advanced diagnostic techniques, as standard therapies may prove ineffective against infections unresponsive to conventional treatments [6].

One of these rare pathogens is *Toxocara canis*, a parasite commonly found in dogs that can infect humans through oral transmission, leading to visceral larva migrans. While toxocariasis most commonly affects the liver and eyes, it can also manifest in the lungs, causing eosinophilic pneumonia [7]. Documented cases of eosinophilic pneumonia due to *Toxocara canis* are rare, and statistical data on incidence remain limited. The few documented cases emphasize the complexity of diagnosing this condition, particularly in immunocompetent patients [8,9].

This publication presents a unique case of a 24-year-old woman with congenital Kartagener syndrome who was diagnosed with eosinophilic pneumonia caused by *Toxocara canis* infection. This is the first case reported in the literature of pulmonary toxocariasis in a patient with primary ciliary dyskinesia. This case not only illustrates the complexity of the health problems that PCD patients may face but also highlights the necessity of considering atypical pathogens in the differential diagnosis of lung infections in these patients.

## 2. Case Presentation

**Figure 3:**
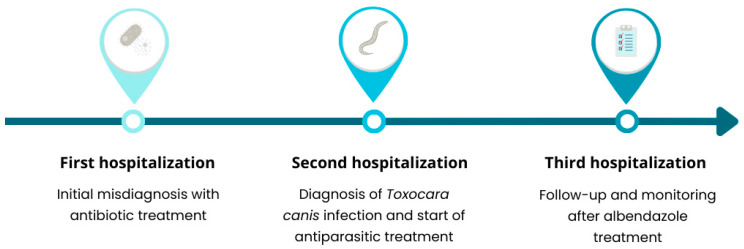
Timeline of hospitalization episodes.

A 24-year-old patient with Kartagener syndrome was admitted to the hospital with symptoms of fluid in the right pleural cavity, confirmed by ultrasound examination. Her medical history included the removal of a middle lobe of the left lung, surgery for hydronephrosis of the right kidney, situs inversus (resulting in a 3-lobe left lung and a 2-lobe right lung), arterial hypertension, and two previous infections with *Toxocara canis.* A week earlier, the patient had completed a two-week hospitalization due to hypertension and chest pain. At that time, due to elevated CRP and signs of a urinary tract infection (UTI), she was treated with amoxicillin and clavulanic acid. Despite the treatment, her symptoms did not resolve, and tests showed persistent eosinophilia. The patient regularly attends specialist check-ups and occasionally requires antibiotic therapy. During the current hospitalization, three pleural punctures were performed. Due to persistent eosinophilia and elevated IgE levels, parasitological diagnostics were conducted, which confirmed *Toxocara canis* infection. Treatment with albendazole was initiated (Figure 3). This case highlights the importance of extended diagnostics in patients with Kartagener syndrome, particularly in cases of atypical infections or lack of response to standard treatment.

### 2.1. Childhood Medical History

The patient was born at 37 weeks of gestation with a birth weight of 3400 g and an Apgar score of 9. The mother’s pregnancy was complicated by gestational diabetes and a cytomegalovirus infection. Situs inversus was diagnosed on the third day of life during a hospital stay for a congenital infection. This condition also involved pulmonary inversion, with the patient having a 3-lobe left lung and a 2-lobe right lung. The infantile period was marked by recurrent respiratory infections, resulting in multiple hospitalizations. The patient was diagnosed with Kartagener syndrome at 5 months of age during hospitalization for pneumonia caused by *Pseudomonas aeruginosa*. A bronchoscopy at the time revealed immobile cilia, reversed bronchial tree arrangement, and abundant mucus secretion. In subsequent years, the girl’s condition remained stable, with only mild infections occurring. Recurrent pneumonias began to appear during adolescence. At the age of 14, the patient was hospitalized due to prolonged pneumonia. At that time, an infection with *Mycoplasma pneumoniae* was diagnosed, along with coexisting parasitic infections caused by *Toxocara canis* and *Ascaris lumbricoides.* During this hospitalization, cirrhotic changes in the middle lobe of the left lung were found, which resulted in the need for a middle lobectomy that same year. Reinfection with *Toxocara canis* and *Ascaris lumbricoides* was diagnosed less than 2 years later when the patient presented with symptoms of exudative pleuritis. An ultrasound of the pleural cavity revealed the presence of 150 mL of free fluid in the left pleural cavity. Additionally, hydronephrosis of the right kidney was noted, which had been operated on when the patient was 15 years old.

### 2.2. First Episode of Hospitalization—Misdiagnosis

Further health problems reappeared at the age of 24, when recurrent respiratory infections occurred again, along with arterial hypertension. As a result, the woman presented to the hospital, where a chest X-ray and an ultrasound of the pleural cavity were performed, confirming the presence of fluid in the right pleural cavity (up to a width of 55 mm), reaching the ninth rib (Appendix B). The patient also reported a persistent cough and stabbing pain in the right side of the chest, radiating to the back and worsening with deep breaths. The maximum blood pressure readings were 180/120 mmHg. A series of laboratory tests were conducted, including a bacterial urine culture. A general urinalysis revealed the presence of numerous bacteria in the urine sediment. The CRP concentration in the serum initially measured 39.45 mg/L (immunoturbidimetric test, Integra 400 plus). Based on the diagnostic X-ray and ultrasound results, as well as the elevated CRP levels and signs of a urinary tract infection (UTI) in the urine, a decision was made to administer antibacterial treatment with amoxicillin and clavulanic acid in the form of injections, 1000 mg + 200 mg, 5 vials (1 vial per day). This decision was made without receiving the results of the bacterial culture. Despite the lack of significant improvement in the patient’s condition, elevated blood parameters (including eosinophilia, 1.15 K/μL), and the absence of follow-up diagnostic tests for the presence of fluid in the pleural cavity, the patient was discharged home. The patient was instructed to continue antibiotic therapy in the form of coated amoxicillin and clavulanic acid tablets, 1000 mg (875 mg + 125 mg) once daily for the next 5 days. Additionally, probiotic support with Enterol (*Saccharomyces boulardii*) was prescribed, as well as antihypertensive medications (amlodipine 10 mg once daily, methyldopa 250 mg three times daily, indapamide 1.5 mg once daily, nebivolol 5 mg once daily).

### 2.3. Second Episode of Hospitalization—Diagnosis of Toxocara canis Infection

**Figure 4:**
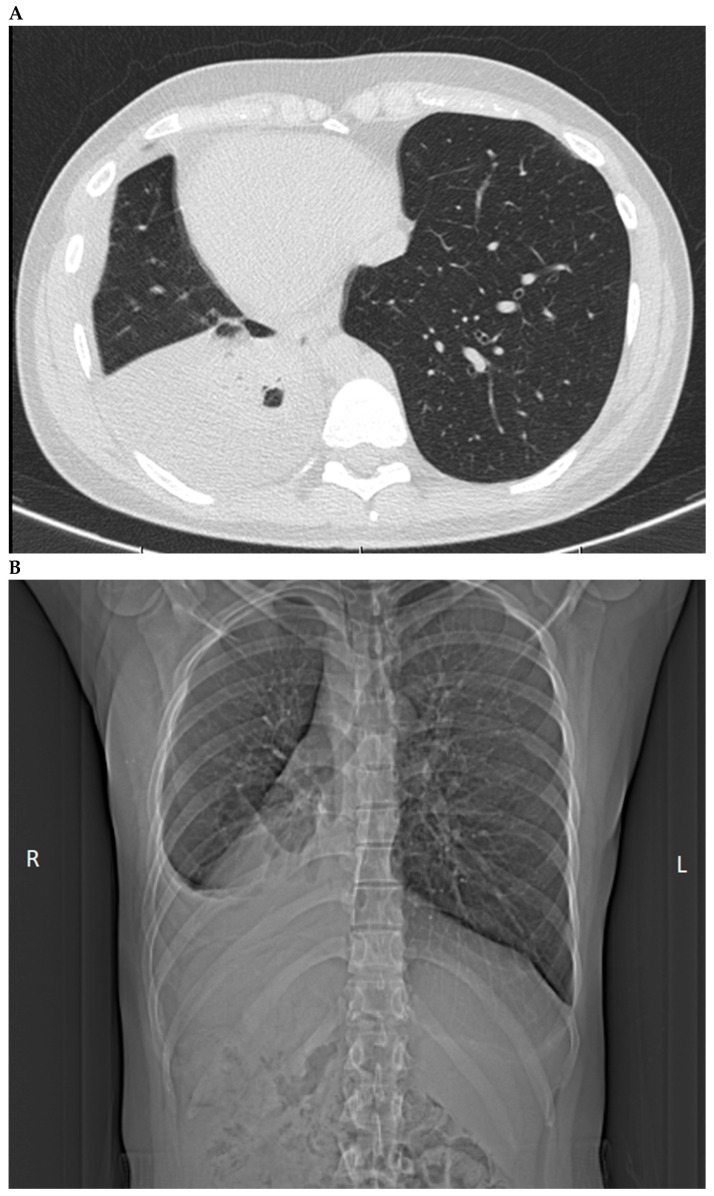
(**A**,**B**) Chest CT images showing a significant presence of fluid in the right lung, taken during the second hospitalization. There is also evidence of situs inversus in the patient (detailed description in Appendix B).

Due to worsening cough, shortness of breath, and a general decline in her health, the patient was readmitted to the hospital seven days after being discharged. Two anaerobic bacteriological cultures of pleural fluid were performed, and after five days of incubation, no microorganisms were grown. Ultrasound of the pleural cavity confirmed a persistent significant amount of fluid (up to 45 mm in width) in the right pleural cavity. Additionally, a non-contrast chest CT scan was performed (Figure 4), which also revealed the presence of fluid in the right pleural cavity (up to 40 mm) (Appendix B). Consolidated band-like opacities were observed in the lower lobe of the right lung, above the fluid, which was consistent with atelectasis and residual inflammatory changes. Situs inversus of the viscera and heart was also confirmed. Laboratory tests showed persistently elevated CRP levels (25.18 mg/L) and eosinophil counts (1.58 K/μL, 18%, reference value: 0.1–0.5 K/μL, 1–5%), along with a high concentration of total IgE (111.0 IU/mL, electrochemiluminescence method, Cobas). It should be noted that the eosinophil count was even higher than during the patient’s first hospital stay. Due to the elevated eosinophil and total IgE levels, tests for parasitic infections were conducted. Stool parasitology did not reveal the presence of gastrointestinal parasites. An ELISA serological test (NovaTec) for *Toxocara canis* IgG antibodies was performed, and the serum showed an elevated sIgG concentration for *Toxocara canis* (16.83 NTU), indicating infection with this canine roundworm. The correlation between elevated blood parameters (*Toxocara canis* sIgG, total IgE, and eosinophils) and abnormalities observed in lung and pleural X-ray imaging led to the correct diagnosis: pleural effusion, not elsewhere classified (ICD-10 code: J90), associated with Löffler’s syndrome (eosinophilic pneumonia caused by toxocariasis). The patient was treated with albendazole, an antiparasitic medication, at a dose of 400 mg/20 mL in the form of an oral suspension. During hospitalization, the patient received three doses of albendazole (once daily). She was discharged with an initial good tolerance of the treatment, with instructions to continue antiparasitic therapy for four more days (400 mg/20 mL albendazole, every morning) and to maintain her antihypertensive regimen as per the initial discharge instructions.

### 2.4. Third Episode of Hospitalization—Control of the Effects of Antiparasitic Treatment 

**Figure 5:**
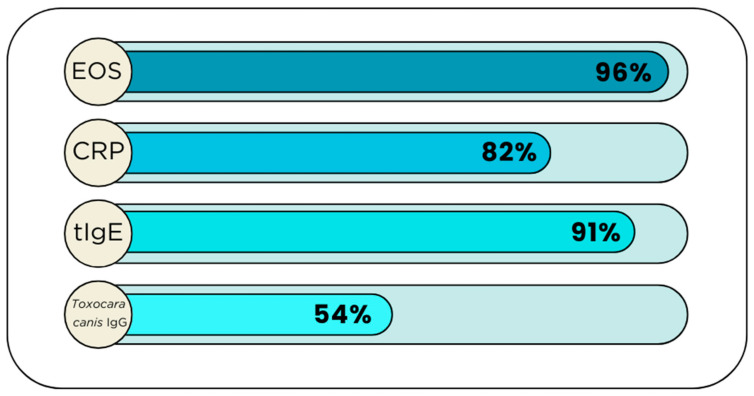
Percentage decrease in inflammatory and parasitic parameters after albendazole treatment (EOS—eosinophils, CRP—C-Reactive Protein, tIgE—total IgE).

Two months after the diagnosis and initiation of albendazole treatment, follow-up biochemical parameters and a chest X-ray were performed. No pleural fluid was detected (Appendix B). A decrease in eosinophil count (0.6%), CRP levels (4.63 mg/L), and *Toxocara canis* IgG antibodies (9.1 NTU) was observed (Table 1, Figure 5). All laboratory results from the three consecutive hospitalizations are summarized in Appendix A. Blood pressure readings were normal, averaging 110/75 mmHg. The patient was referred to an infectious diseases clinic for ongoing care and, in good condition, was discharged with the recommendation to continue antihypertensive medication as per the initial discharge plan. Currently, the symptoms of acute pneumonia have resolved, indicating effective antiparasitic therapy for *Toxocara canis*-induced pneumonia (Löffler’s syndrome) in the patient, who also has Kartagener syndrome.

## 3. Discussion

The clinical case of our patient demonstrates a rare combination of *Toxocara canis* infection with Kartagener syndrome, leading to eosinophilic pneumonia. This atypical clinical manifestation requires a detailed analysis of the interaction between toxocariasis pathogenesis, and the disorders related to Kartagener syndrome, in the context of current scientific and epidemiological knowledge.

Toxocariasis is a relatively common zoonotic parasitic disease. It is estimated that the seroprevalence of *Toxocara* spp. infections in Europe is 11%, while in Poland, this rate reaches 16%. Additionally, approximately 20% of Polish soils are contaminated *with Toxocara* spp. eggs [10]. Despite its prevalence, the manifestation of toxocariasis as pneumonia is rare. Typically, hepato- and splenomegaly, neurotoxocariasis, and ocular toxocariasis are observed [11]. A search of the PubMed database yielded only nine case studies (from 1994 to 2024) describing pneumonia cases caused by *Toxocara canis* (keywords: pneumonia, *Toxocara canis*, case study). This makes the described case particularly interesting from a clinical, diagnostic, and scientific perspective.

The key diagnostic parameters were:

- High antibody titer against *Toxocara canis* (1:800);
- Significant eosinophilia (18% in the peripheral blood smear);
- Elevated total IgE levels;
- Characteristic chest X-ray changes.

The primary parameter identifying *Toxocara canis* infection is the detection of sIgG antibodies. However, due to the long circulation of specific antibodies and possible cross-reactions with other nematodes, it is also recommended to assess eosinophil and total IgE levels to detect active infection. The correlation between *Toxocara canis* infection and elevated total IgE and eosinophil levels has been confirmed in many scientific studies [12,13].

The increase in eosinophil levels in alveoli and lung interstitial tissue is a common marker of the heterogeneous group of diseases known as eosinophilic pneumonias [14]. Eosinophilic pneumonia can occur as a result of autoimmune, neoplastic, or inflammatory diseases, certain medications, and parasitic infections [15]. In this case, eosinophilia (1.15–1.58 K/µL) fell within the mild (0.5 to 1.5 K/µL) to moderate (1.5–5.0 K/µL) range, which is typical for parasitic infections [16]. This increase in eosinophils, along with elevated total IgE levels, reflects the body’s specific immune response to the presence of *Toxocara* larvae. These observations align with studies by Magnaval et al. [17] and Fillaux and Magnaval [18], who emphasized the importance of these parameters in diagnosing toxocariasis.

The pathogenic mechanism in this case can be classified as Löffler’s syndrome, an eosinophilic pneumonia resulting from the trans-pulmonary migration of *Toxocara* larvae. This process involves the migration of larvae to lung tissue, a local inflammatory response with eosinophil infiltration, mast cell activation, the release of inflammatory mediators, and IgE antibody production leading to mast cell degranulation [19,20]. These immunological processes, as described in detail by Pinelli et al. [21], combined with the impaired ciliary function characteristic of Kartagener syndrome, led to an intensified inflammatory response in the patient’s lungs. Kartagener syndrome, a rare autosomal recessive genetic disorder, played a key role in the progression of the infection. The characteristic triad of Kartagener syndrome involving situs inversus, bronchiectasis, and chronic sinusitis, created favorable conditions for parasite colonization and dissemination in the patient’s lungs [22]. The dysfunction of respiratory cilia, typical of Kartagener syndrome, likely facilitated the settlement of *Toxocara canis* larvae in lung tissue, a phenomenon that is usually rare. Notably, bronchiectasis and chronic inflammation, present in Kartagener syndrome, may have created particularly conducive conditions for the development of *Toxocara* infection, leading to more severe eosinophilic pneumonia than typically observed in toxocariasis cases. This observation is consistent with the studies by Bede et al., who demonstrated increased susceptibility to infections in patients with primary ciliary dyskinesia [23]. While PCD itself does not increase susceptibility to *Toxocara canis* infection through an immunodeficiency mechanism, the impaired mucociliary clearance characteristic of PCD can exacerbate the severity of respiratory symptoms if such an infection occurs. Specifically, the reduced capacity to effectively clear mucus and pathogens from the respiratory tract in PCD patients allows parasites like *Toxocara canis* to persist longer within lung tissue, creating an environment that may intensify the local inflammatory response [24]. This prolonged presence of pathogens due to compromised airway clearance means that, although PCD patients do not face an elevated risk of acquiring *Toxocara canis*, they may experience a more severe progression of respiratory symptoms and inflammation once the infection is established.

The uniqueness of our case warrants comparison with existing literature, although documented cases of pulmonary toxocariasis in patients with primary ciliary dyskinesia (PCD) are exceptionally rare, limiting comprehensive comparative analysis. While pulmonary toxocariasis has been reported in various respiratory conditions, the combination with PCD represents a notably unusual clinical scenario [25,26]. Our observations suggest that while PCD may not increase susceptibility to Toxocara infection per se, the pre-existing impairment of mucociliary clearance can substantially amplify respiratory manifestations. This finding aligns with research by Ranasuriya et al. (2016), who documented atypical presentations of pulmonary toxocariasis in immunocompromised patients, highlighting how underlying conditions can modify disease expression [27]. Interestingly, contrary to Gonzalez-Quintela et al. [28], we observed no significant spirometric changes attributable to Toxocara infection, potentially due to the overshadowing effects of Kartagener syndrome on pulmonary function parameters.

A noteworthy aspect of our case was the elevation in hepatic enzymes (ALT, AST) following therapeutic intervention with albendazole. This observation adds to the existing literature documenting transient hepatic enzyme elevation during albendazole treatment [29]. While the precise mechanisms of albendazole-induced liver function alterations remain incompletely understood, current evidence suggests these changes are typically transient, with normalization expected post-treatment [29]. This emphasizes the importance of systematic liver function monitoring throughout the therapeutic course.

In addressing the Toxocara canis-induced eosinophilic pneumonia in our patient, albendazole was selected as the primary therapeutic agent based on its established antiparasitic efficacy. The observed clinical improvement and reduction in inflammatory markers validated this therapeutic choice. For cases presenting with severe inflammatory responses, particularly in the context of PCD where compromised airway clearance may intensify inflammation, a dual therapeutic approach incorporating both antiparasitic treatment and anti-inflammatory measures may be optimal. The potential benefit of adjunctive corticosteroid therapy in such cases, supported by the current literature [30,31], lies in its ability to modulate the inflammatory response while albendazole addresses the underlying parasitic infection. This comprehensive treatment strategy may be particularly valuable in complex cases where both infection control and inflammation management are crucial for optimal outcomes.

**Figure 6:**
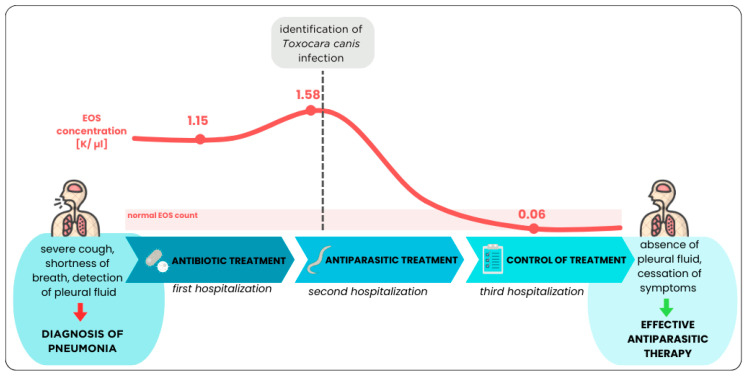
Effect of the treatment type on eosinophil concentration and the course of the disease.

It is also worth noting that antibiotic therapy was initially administered, which is the most common approach to treating pneumonia. Bacteria, particularly *Streptococcus pneumoniae*, are responsible for the majority of pneumonia cases. Before the widespread use of antibiotics, *Streptococcus pneumoniae* was responsible for 95% of pneumonia cases [32]. Although bacterial causes are still the most common (24% of bacterial pneumonias in Europe), the etiology of acute pneumonia remains unidentified in about 60% of cases [33]. In this case, despite elevated eosinophil levels, which often indicate an allergic or parasitic infection, antibiotic therapy was also administered, but it did not yield the expected results (Figure 6). This situation underscores the importance of thorough differential diagnosis in cases of atypical pneumonia, especially in patients with rare genetic syndromes.

Potential long-term consequences of this case should also be considered. Kartagener syndrome itself predisposes patients to recurrent respiratory infections and may lead to permanent lung damage [34]. The superimposition of *Toxocara canis* infection may have accelerated these processes. Therefore, the patient will require special care and monitoring in the future. For patients diagnosed with PCD, preventive measures include maintaining rigorous hygiene practices, routine check-ups, relevant physiotherapy interventions, and careful monitoring for symptoms that may indicate atypical infections. Regular follow-up and immediate intervention in the case of respiratory symptoms can reduce the risk of secondary infections [35,36].

This case also highlights the need for further research into the interactions between congenital respiratory disorders and parasitic infections. It would be particularly interesting to investigate whether patients with Kartagener syndrome are more susceptible to atypical manifestations of parasitic infections and whether these infections have long-term effects on the course of their underlying disease.

This case underscores the importance of differential diagnosis in atypical infections for patients with PCD. Key limitations include the lack of extensive data on parasitic infections within PCD populations, as most studies focus on bacterial and viral pathogens rather than parasitic agents. Additionally, the rarity of both primary ciliary dyskinesia and pulmonary toxocariasis complicates the generalizability of findings, as cases with concurrent presentation of these conditions are infrequent and underreported. The absence of large-scale studies limits our ability to draw definitive conclusions about the typical progression, optimal diagnostic approaches, and specific treatment efficacy for parasitic infections in PCD patients. Further research, including prospective studies and broader case series, is necessary to clarify the interactions between PCD and atypical infections such as toxocariasis, ultimately guiding more precise diagnostic and therapeutic strategies.

## 4. Conclusions

The occurrence of diseases that impair lung defense mechanisms, such as primary ciliary dyskinesia (PCD), increases the likelihood of parasitic larvae, like *Toxocara canis*, invading and proliferating in lung tissue. In cases where pneumonia-like symptoms arise in PCD patients, parasitic infection should be considered as a potential cause. Elevated eosinophil counts, total IgE levels, and specific IgG antibodies in the blood can be indicative of a *Toxocara canis* infection. Early detection of parasitic infections allows for timely treatment, significantly reducing the risk of complications that may arise from prolonged illness and pose a threat to the patient’s health.

## Figures and Tables

**Table 1:** Presentation of key laboratory parameters indicative of toxocariasis before and after appropriate therapy.

Laboratory Findings	First Hospitalization— Misdiagnosis	Second Hospitalization— *Toxocara canis* Detection	Third Hospitalization— Control of Treatment	Reference Interval
Eosinophils	1.15 (15.4)	1.58 (18.0)	0.06 (0.6)	0.1–0.5 K/µL (1–5%)
CRP	14.13	25.18	4.63	0–5 mg/L
IgE total	-	111.0	9.7	0–100 IU/mL
*Toxocara* *canis* IgG	-	16.83	9.1	>9 NTU negative 9–11 inconclusive <11 positive

increased parameter, decreased parameter.

## Data Availability

The data supporting the findings of this study are derived from clinical observations and the medical records of the patient discussed in this case report. Due to privacy and ethical considerations, these data are not publicly available. Any additional information or details that do not compromise patient confidentiality can be provided upon reasonable request to the corresponding author, Kacper Packi, and subject to approval from the relevant ethical review board.

## Appendix A. Laboratory Test Results

**Laboratory** **Findings**	**4 April 2024**	**10 April 2024**	**19 April 2024**	**26 April 2024**	**1 May 2024**	**24 June 2024–28 June 2024**	**Reference** **Interval**
	**Start of Antibiotic Therapy** **(First Hospital Stay)**		**Identification of Toxocariasis and** **Implementation of Appropriate** **Treatment (Second Hospital Stay)**			**Control of Treatment** **(Third Hospital Stay)**	
**Blood morphology**							
Leukocytes	10.2	7.5	8.8	-	6.8	10.1	3.8–10 K/uL
Erythrocytes	5.3	5.44	5.55	-	5.4	5.09	3.7–5.1 mln/uL
Hemoglobin	15.6	15.8	16.2	-	15.6	15.0	12–16 g/dL
Haematocrit	43.5	45.3	44.9	-	44.4	43.7	37–47%
Blood platelets	225	278	264	-	258	233	140–440 K/mL
Neutrophils	-	3.70	4.70	-	-	7.33	2.5–7 K/uL
Lymphocytes	-	1.77	1.6	-	-	1.93	1–3.5 K/uL
Monocytes	-	0.83	0.87	-	-	0.76	0.2–1 K/uL
Eosinophils	-	1.15	1.58	-	-	0.06	0.1–0.5 K/uL
Basophiles	-	0.0	0.0	-	-	0.0	0–0.1 K/uL
Neutrophils	-	49.4	53.6	-	-	72.3	40–70%
Lymphocytes	-	23.7	18.2	-	-	19.1	20–45%
Monocytes	-	11.1	9.9	-	-	7.5	2–10%
Eosinophils	-	15.4	18.0	-	-	0.6	1–5%
Basophiles	-	0.4	0.3	-	-	0.5	0–2%
**Biochemistry**							
*Toxocara canis* IgG	-	-	-	16 . 83	-	9.1	>9 NTU—negative 9–11 inconclusive <11 positive
LDH	-	-	228	-	-	-	225–450 U/L
Total protein	-	-	7.1	-	-	-	6.4–8.3 g/dL
Total bilirubin	0.75	-	0.66	-	-	1.22	0–0.9 mg/dL
Glucose	96	-	95	-	-	93	70–99 mg/dL
GGTP	-	-	9	-	-		6–42 U/L
Urea	-	-	46.3	-	33.4	38.6	16.6–48.5 mg/dL
Sodium	136	-	140	-	141	138	136–145 mmol/L
Potassium	3.52	-	3.47	-	4.05	3.69	3.5–5.1 mmol/L
Magnesium	0.91	-	-	-	-	-	0.66–1.07 mmol/L
ALT	11	-	12	-	-	140	0–33 U/L
AST	17	-	17	-	-	86	0–32 U/L
CK-MB	-	-	-	-	-	10.2	0–25 U/L
Troponin I	<0.100	-	-	-	-	<0.100	0–0.3 ng/mL
NT-proBNP	33.44	-	21.99	-	-	-	0–125 pg/mL
Creatinine	0.87	-	1.06	-	1.13	0.98	0.5–0.9 mg/dL
eGFR	80.0		63.7	-	59.2	69.7	<90 mL/min/1.73 m^2^—normal 60–89—mildly reduced 30–59 moderately reduced 15–29 severely reduced >15 renal failure
**Immunochemistry**							
CRP	39.45	14.13	25.18	-	5.12	4.63	0–5 mg/L
Total IgE	-	-	111.0	-	-	9.7	0–100 IU/mL
**Hormones**							
TSH	2.120	-	2.270	-	-	0.720	0.27–4.2 uIU/mL (non-pregnant women)
ft3	3.07	-	-	-	-	-	2.04–4.04 ng/dL (non-pregnant women)
ft4	1.28	-	-	-	-	-	0.93–1.7 ng/dL (non-pregnant women)
**Arterial Blood Gas**							
pH	7.44	-	7.48	-	-	7.42	7.35–7.45
pCO_2_	25.4	-	34.00	-	-	32.5	35–45 mmHg
pO_2_	68.6	-	62.00	-	-	60.3	80–100 mmHg
Saturation	94.7	-	93.40	-	-	91.8	90–96%
HCO_3_	17.00	-	24.50	-	-	20.5	20–26 mmol/L
BE	−5.20	-	1.50	-	-	−3.00	−2.5–5.5 mmol/L
**Coaguology**							
D-dimers	1.56	-	-	-	-	0.41	0–0.5 ug/mL
INR	0.99	-	1.16	-	-	1.09	0.8–1.2
Prothrombin index	101	-	87	-	-	92	80–120%
Prothrombin time	10.5	-	12.2	-	-	11.3	8–12 s
increased parameter, decreased parameter.							

## Appendix B. Description of Diagnostic Imaging Examinations

The patient presents with situs inversus, involving pulmonary inversion, with a 3-lobe left lung, 2-lobe right lung, right-sided heart and aortic arch, liver on the left side, and spleen on the right side.

**Type of Examination**	**First Hospitalization**	**Second Hospitalization**	**Third Hospitalization**
Chest X-ray	5 April 2024: Fluid in the right pleural cavity, extending up to the ninth rib and entering the oblique fissure. Pulmonary field without focal lesions. Cardiac silhouette within normal limits.	22 April 2024: In the right pleural cavity, fluid up to 29 mm in width is present. No fluid observed in the left pleural cavity. 26 April 2024: In the right pleural cavity, fluid up to 45 mm in width is present. No fluid is observed in the left pleural cavity.	24 June 2024: No signs of fluid were observed in the right or left pleural cavity.
Abdominal ultrasound	5 April 2024: Situs inversus. The liver demonstrates normal echogenicity, is homogeneous, with no focal lesions. The gallbladder has thin walls and is free of stones. The pancreas, within the scope of the examination, is homogeneous and not enlarged. The spleen is homogeneous and not enlarged. The kidneys are of normal shape, size, and position, with no signs of nephrolithiasis. The right kidney shows an enlarged pelvicalyceal system (PCS), with calyces up to 10 mm in width and the renal pelvis up to 27 mm in width. The abdominal aorta, within the scope of the examination, shows no pathological widening of the lumen. No free fluid is observed in the peritoneal cavity. The urinary bladder is empty and cannot be assessed.	-	-
Pleural cavity ultrasound	8 April 2024: In the right pleural cavity, fluid up to 55 mm in width is present. No fluid was observed in the left pleural cavity.	-	-
Chest CT (without contrast enhancement)	-	23 April 2024: Exudative pleurisy of the right pleural cavity, associated with Löffler’s syndrome (eosinophilic pneumonia in the context of toxocariasis). Situs inversus with pulmonary inversion. The heart and aortic arch are on the right side. The liver is on the left side, and the spleen is on the right. Fluid is present in the right pleural cavity, up to 40 mm in thickness. It was noted that there are atelectatic/inflammatory changes in the lower lobe of the right lung with an accompanying layer of fluid in the right pleural cavity. No enlarged lymph nodes in the mediastinum. The heart is not enlarged, there is no fluid in the pericardial sac, and the aorta is not dilated. The right kidney shows a segmentally thinned cortical layer with hypodense areas, most likely cysts. Bone structures show no pathological changes.	-
